# Defining the Cause of Death in Hospitalised Patients with Acute Kidney Injury

**DOI:** 10.1371/journal.pone.0048580

**Published:** 2012-11-02

**Authors:** Nicholas M. Selby, Nitin V. Kolhe, Christopher W. McIntyre, John Monaghan, Nigel Lawson, David Elliott, Rebecca Packington, Richard J. Fluck

**Affiliations:** 1 Department of Renal Medicine, Royal Derby Hospital, Derby, United Kingdom; 2 School of Graduate Entry Medicine and Health, University of Nottingham, Nottingham, United Kingdom; 3 Department of Chemical Pathology, Royal Derby Hospital, Derby, United Kingdom; 4 Department of Informatics, Royal Derby Hospital, Derby, United Kingdom; University of Sao Paulo Medical School, Brazil

## Abstract

**Background:**

The high mortality rates that follow the onset of acute kidney injury (AKI) are well recognised. However, the mode of death in patients with AKI remains relatively under-studied, particularly in general hospitalised populations who represent the majority of those affected. We sought to describe the primary cause of death in a large group of prospectively identified patients with AKI.

**Methods:**

All patients sustaining AKI at our centre between 1^st^ October 2010 and 31^st^ October 2011 were identified by real-time, hospital-wide, electronic AKI reporting based on the Acute Kidney Injury Network (AKIN) diagnostic criteria. Using this system we are able to generate a prospective database of all AKI cases that includes demographic, outcome and hospital coding data. For those patients that died during hospital admission, cause of death was derived from the Medical Certificate of Cause of Death.

**Results:**

During the study period there were 3,930 patients who sustained AKI; 62.0% had AKI stage 1, 20.6% had stage 2 and 17.4% stage 3. In-hospital mortality rate was 21.9% (859 patients). Cause of death could be identified in 93.4% of cases. There were three main disease categories accounting for three quarters of all mortality; sepsis (41.1%), cardiovascular disease (19.2%) and malignancy (12.9%). The major diagnosis leading to sepsis was pneumonia, whilst cardiovascular death was largely a result of heart failure and ischaemic heart disease. AKI was the primary cause of death in only 3% of cases.

**Conclusions:**

Mortality associated with AKI remains high, although cause of death is usually concurrent illness. Specific strategies to improve outcomes may therefore need to target not just the management of AKI but also the most relevant co-existing conditions.

## Introduction

Acute Kidney Injury (AKI) is an important condition affecting up to 22% of hospitalised patients [1], [2]. The poor outcomes and high mortality rates associated with this condition have been well described in many different clinical settings [1], [3], [4], [5], [6], [7]. Despite this, there are very few studies that specifically report causes of death (CoD) in patients with AKI. Those that do include small patient numbers, are confined to specific patient groups (often intensive care) or predate current diagnostic criteria by several decades [8], [9], [10], [11]. For the latter studies this meant that only patients with severe AKI were studied who are not representative of a more generalised population. In addition, there may be differences between historical treatment patterns as compared to current practice. The study of CoD in patients with AKI has relevance as AKI rarely occurs in isolation; co-existing conditions are often implicated in its aetiology as well as having a strong impact on patient outcome [11]. In addition, it is increasingly appreciated that AKI has distant organ effects that contribute to organ dysfunction and impact on overall outcomes [12].

Currently, there are no specific therapies that are effective for AKI, and attempts to improve outcomes have focussed on improving basic elements of AKI management [13]. However, it seems plausible that to effect meaningful improvements, the development of successful strategies should encompass the relevant co-existing conditions and organ dysfunction as well as the management of AKI. Some focus may be provided by identification of the most common CoD in patients with AKI. We therefore performed a prospective observational study to report CoD in patients with AKI from a generalised hospital population. We also sought to confirm factors associated with mortality in this group.

## Methods

### Setting

The Royal Derby Hospital is a 1,139 bedded teaching hospital that provides all major medical and surgical specialties (excepting neuro- and cardiothoracic surgery). The renal unit is a tertiary referral centre, serving a population of 700,000. There is a central chemical pathology laboratory for all inpatient and outpatient samples. A compensated kinetic Jaffe method with an inter-assay coefficient of variance of 2.3% at 96 µmol/l (Roche P-analyser, RocheDiagnostics, W. Sussex, UK) was used to measure all serum creatinine values throughout the study period (normal creatinine range 70–120 µmol/l).

### Electronic Reporting System for AKI

We have developed a real-time, hospital-wide, electronic reporting system for AKI based on the Acute Kidney Injury Network (AKIN) diagnostic criteria [14]. This system has been in clinical use since 2010 and its diagnostic accuracy has been established; it also allows prospective data capture for all cases of AKI at our centre. A full description of the system is available elsewhere [15] but in brief, all serum creatinine measurements sent from in-patient locations are included, with the exception of those from the renal dialysis unit and renal inpatient ward. The pathology computer system (iLab 5.8.1001, iSoft) automatically compares all measured creatinine values on an individual patient basis against an estimated baseline creatinine reverse calculated from the MDRD equation assuming a glomerular filtration rate (GFR) of 75 ml/min/1.73 m^2^ [16]. All measured creatinine values that are 50% greater (1.5x) than the individual’s estimated baseline value are flagged internally within iLab. These results are then reviewed by a clinical chemist, who selects the real baseline creatinine for each patient using previous creatinine results and applies the AKIN criteria (accepting the estimated baseline from reverse MDRD calculation in those cases where there were no previous creatinine measurements available). For each acute elevation in creatinine consistent with AKI a report is issued in the hospital results reporting system (Clinical Manager 1.5, iSoft) that specifies AKI stage, value and date of baseline creatinine employed, an intranet link to local AKI clinical guidelines and a reminder of the AKIN diagnostic criteria.

This system allows prospective data collection for all cases of AKI at our centre. A daily electronic report of all AKI episodes is automatically generated that details patient location (community versus hospital-acquired), patient age, and baseline creatinine. These data are supplemented by highest AKI stage, last serum creatinine in stay (to assess renal recovery), length of hospital stay and whether the patient survived to hospital discharge. Co-morbidity (based on ICD-10 coding) and Charlson score are also included. Approval to use anonymised data in this way was obtained from the National Information Governance Board. Patients who did not sustain AKI were not included.

### Cause of Death

For those patients with AKI that died, CoD was taken from an internal database that records details from all Medical Certificates of Cause of Death (MCCD), the completion of which is governed by UK law. At our centre, a policy exists to ensure consistency in MCCD completion. Certain deaths must be reported to HM Coroner (those which were violent, sudden or with an unknown cause); in other cases in which the CoD was apparent the MCCD is issued directly. For the latter group, the certificate is completed by doctor with a minimum of one year of experience and the CoD is determined after discussion with the attending physician in charge of the case. For those cases that are examined by HM Coroner, CoD are issued after a postmortem examination or after the completion of an inquest.

Immediately after the completion of the MCCD the CoD are sent to a clinical auditor who enters these into an electronic database. CoD for patients who subsequently undergo postmortem examination are also collated. To ensure data quality, the CoD database is cross-checked monthly against an automatically generated list of all in-hospital deaths to identify cases in which CoD is missing; data for these cases is sourced from the MCCD records or notification of death correspondence. CoD summary data are also generated and reviewed on a monthly basis by a committee of senior doctors representing all major specialities. This group identifies anomalies in MCCD CoD results, can review specific cases and then correct entries in CoD database as appropriate.

### Statistical Analysis

Parametric data are presented as mean±standard deviation and non-parametric data as median (inter-quartile range). Chi-squared test was used to compare categorical data and t-test or Mann-Whitney test to compare continuous data depending on whether data were parametric or non-parametric. Binary logistic regression was used to test significant univariate associations with in-hospital mortality. In-hospital mortality was chosen as CoD were only available for patients who died in hospital. P-values of <0.05 were considered significant. All analyses were performed using SPSSv.19.

## Results

From 1^st^ October 2010 until 31^st^ October 2011 there were 3,930 patients who sustained AKI. 2437 patients (62.0%) had AKI stage 1, 811 (20.6%) patients stage 2 and 682 (17.4%) stage 3. Overall in-hospital mortality rate was 21.9% (859 patients) and mortality rate at 30 days post AKI was 23.8% (931 patients). Median age was 80 years (IQR 16) and the vast majority of patients (3634, 92.5%) were admitted to hospital as an emergency. 2558 (65.1%) patients had community acquired AKI with the remainder sustaining AKI during their hospital admission. 68% of patients had a Charlson index of one or greater and median score was 1 (IQR 3). The proportion of patients receiving RRT was low at 2.5% (99 patients). Males and females were equally represented (1947 patients were male, 49.5%) and reflecting our centre’s catchment population, 3532 (89.9%) of patients were Caucasian.

### Causes of Death

CoD could be identified in 802 cases (93.4%). Reasons for unavailability of CoD were that MCCD was issued elsewhere (2 cases, 0.2%), cases still awaiting inquest (23 cases, 2.7%) and missing data in 32 cases (3.7%).

**Figure 1:**
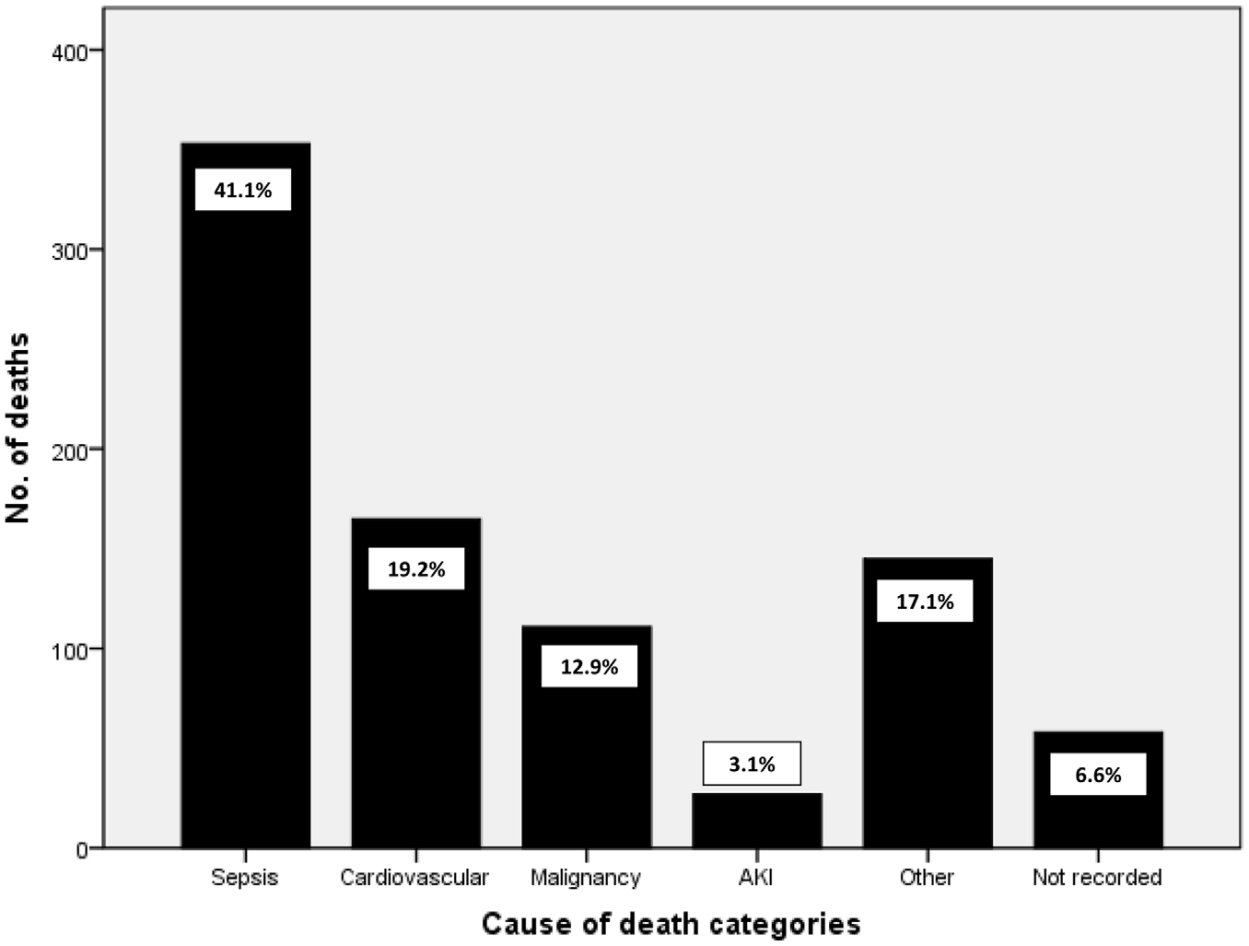
Frequency chart demonstrating major disease categories causing death in patients with AKI.

There were three main disease categories accounting for three quarters of all mortality; sepsis (353 cases, 41.1%), cardiovascular disease (165 cases, 19.2%) and malignancy (111 cases, 12.9%). These data are summarised in figure 1. The major diagnosis leading to sepsis was pneumonia, accounting for a third of all deaths (286 cases, 33.3%) whilst heart failure (84 cases, 9.8%) and ischaemic heart disease (56 cases, 6.5%) accounted for the majority of cardiovascular deaths. Table 1 displays the relative frequency of the most common individual conditions leading to death. The major conditions leading to death displayed a similar pattern and remained fairly constant across the different AKI stages (shown in table 2), although with increasing AKI severity there was a trend to fewer cardiovascular deaths and a greater proportion due to multi-organ failure.

**Table 1:** Table of the most frequent primary causes of death.

Condition leading to death	Number of cases	Percentage of total deaths
Pneumonia	286	33.3%
Malignancy	99	11.5%
Heart failure	84	9.8%
Ischaemic heart disease	56	6.5%
Urinary sepsis	28	3.3%
AKI	27	3.1%
Stroke	21	2.4%
Multi-organ failure	25	2.9%
Ischaemic bowel	21	2.4%
Cirrhosis of the liver	14	1.6%
Pulmonary embolus	14	1.6%
Soft tissue infection	13	1.5%
Perforated abdominal viscus	13	1.5%
Haematological malignancy	13	1.5%
Bowel obstruction	9	1.0%
Ruptured aortic aneurysm	9	1.0%
Septicaemia (unspecified)	8	0.9%
Unavailable	57	6.6%

**Table 2:** Table of the most frequent primary cause of death stratified by AKI stage.

Condition leading to death	AKI stage 1	AKI stage 2	AKI stage 3
Pneumonia	34.1% (138)	35.8% (87)	28% (61)
Malignancy	12.3% (50)	11.6% (28)	15.6% (34)
Heart failure	12.6% (51)	8.2% (20)	6.1% (14)
Ischaemic heart disease	9.4% (38)	3.3% (8)	4.6% (10)
Multi-organ failure	2% (8)	4.1% (10)	4.6% (10)
Total number of deceased	402	241	216

Results are displayed as percentages with absolute numbers in parentheses.

Of those that died, 145 (16.8%) cases underwent post-mortem examination. The frequency of specific CoD in this subset was very similar to the overall group. Pneumonia remained the most common CoD in 49 (33.8%), with cardiovascular disease again being the second most common (27 cases, 18.6%). As previously, cardiovascular disease comprised mainly of ischaemic heart disease (18 cases, 12.4%) and heart failure (7 cases, 4.8%). The third most common CoD was malignancy (9 cases, 6.2%). Results of 23 (15.9%) post-mortem examinations were not available at time of data collection.

AKI was the primary CoD in only 27 (3.1%) cases; 14 of these cases had sepsis as a precipitating cause and 8 were in association with advanced malignancy. Only two MCCD were completed as AKI without a precipitating event. The small proportion of patients with AKI as the primary CoD was consistent with a median final creatinine in non-survivors of 168 µmol/l (IQR 116 µmol/l); 797 (92.8%) of non-surviving patients had a final serum creatinine of <400 µmol/l. Although this demonstrates that the majority of patients did not die as a direct result of uraemia, 580 (76.2%) patients had not recovered renal function by the time of death (renal recovery defined as serum creatinine less than 26 µmol/l above baseline value). In 98 patients, there were no creatinine data to assess recovery.

Despite studying a group of patients who all had AKI and in whom less than a quarter of non-survivors had recovered their baseline renal function, only 102 (11.9%) of the MCCDs included AKI in any section, signifying under-reporting of AKI.

### Factors Associated with Mortality in AKI

**Figure 2:**
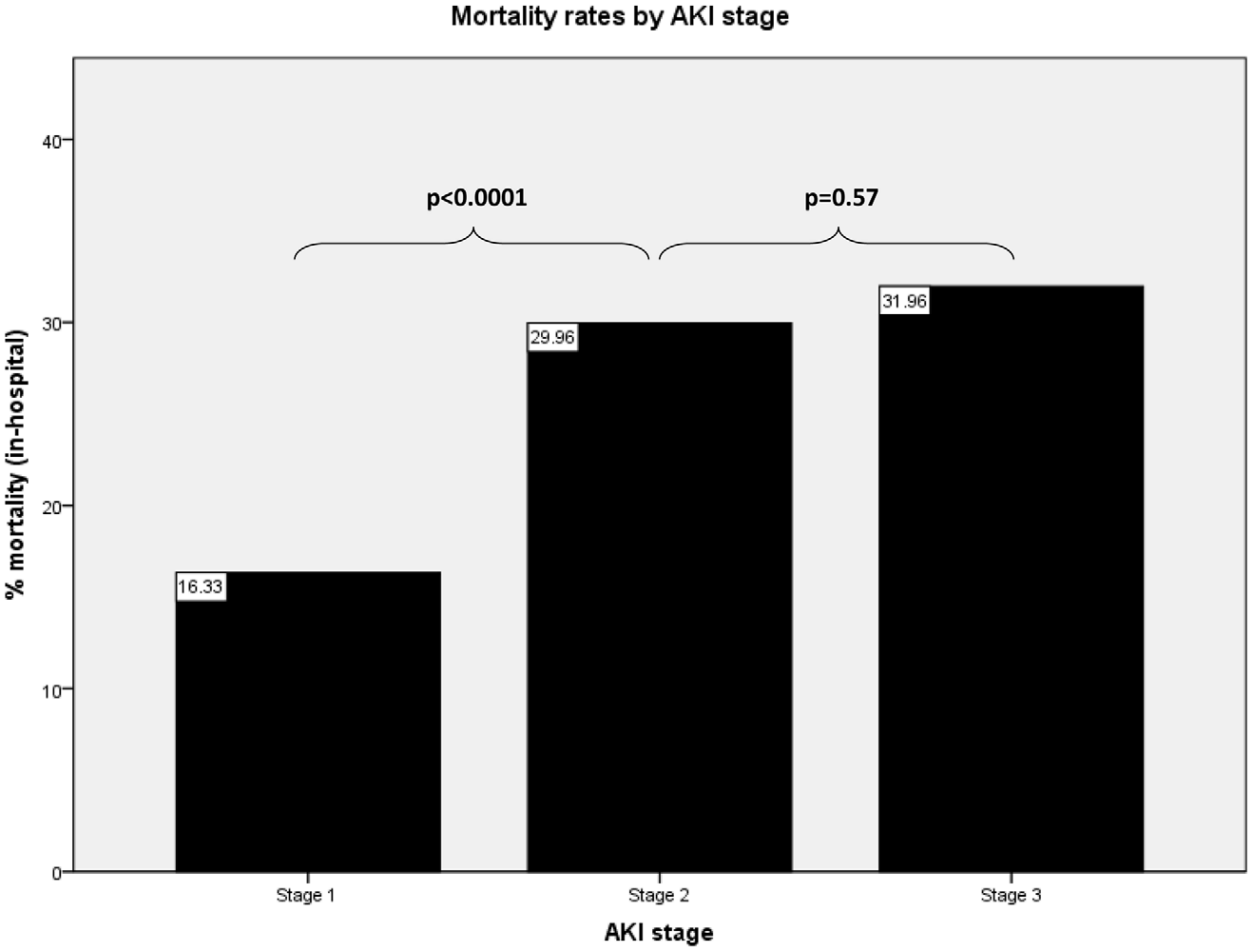
In hospital mortality rates stratified by AKI stage.

We compared survivors and non-survivors to identify factors associated with in-hospital mortality. Mortality rates associated with different AKI stages are summarised in figure 2. The mortality rate in AKI stage 1 of 16.3% was significantly lower than either AKI stage 2 (mortality rate 30%) or stage 3 (mortality rate 32.0%, p<0.0001 for each comparison) whilst mortality rates in stages 2 and stage 3 were similar (p = 0.57) as previously reported [15]. Increasing age was also associated with increased risk of mortality; median age was 79 yrs (IQR 17 ys) in survivors as compared with 82 yrs (IQR 14 yrs) in non-survivors (p<0.0001). Community acquired AKI was associated with a lower mortality rate as compared to AKI occurring after admission to hospital (19.1% versus 26.1%, p<0.0001). Elective admission also conferred a lower mortality rate of 8.8% as compared to those admitted as an emergency in which mortality was 22.7% (p<0.0001). In patients who received RRT mortality was significantly higher as compared to those who did not (43.4% versus 21.3%, p<0.0001). Co-morbid conditions also were associated with mortality and Charlson co-morbidity index was higher in non-survivors (2, IQR 2) as compared with survivors (1, IQR 2, p<0.0001). The specific conditions that had individual associations with mortality are shown in table 3.

**Table 3:** Univariate associations between co-morbid conditions and mortality in patients with AKI.

Co-morbid condition	Odds ratio of in-hospital mortality	95% CI
Acute myocardial infarction	1.3	1.1–1.6
Stroke	1.7	1.1–2.6
Congestive cardiacfailure	2.0	1.7–2.5
Liver disease	2.2	1.1–4.3
Severe liver disease	6.1	3.1–11.8
Peripheral vasculardisease	1.7	1.2–2.5
Cancer	1.6	1.3–2.1
Metastatic disease	3.0	2.3–3.9
Pulmonary disease	1.5	1.2–1.8

There were no associations with diabetes, diabetic complications or dementia.

Binary logistic regression including all significant univariable associations revealed that increasing AKI stage remained associated with in-hospital mortality after accounting for the effects of age and co-morbidity (Nagelkerke r^2^ = 0.14, Hosmer and Lemeshow test for goodness of fit p = 0.08). The full results of this model are displayed in table 4.

**Table 4:** Binary logistic regression model with in-hospital mortality as the dependent factor.

Factor	Hazard ratio of in-hospital mortality	95% CI
Severe liver disease	8.1	3.9–16.8
Metastatic disease	3.6	2.7–4.7
AKI stage 3	3.0	2.4–3.7
AKI stage 2	2.4	2.0–2.9
Congestive cardiac failure	2.0	1.6–2.6
Pulmonary disease	1.5	1.2–1.9
Hospital acquired AKI	1.6	1.4–1.9
Age (per 5 yrs)	1.2	1.1–1.2

Variables with significant associations with mortality were tested; for AKI stage, stage 1 was taken as the reference category. Hospital acquired AKI was compared with community acquired AKI that was used as the reference category.

## Discussion

This study provides a detailed and current description of CoD in a large group of patients with AKI, and is the first to do so in a generalised hospital population in whom current diagnostic criteria are employed. The identification of the most common conditions leading to death may help the design of future strategies to improve the outcomes of AKI that target relevant co-existing pathologies.

There are a huge number of recent studies attesting to the high incidence of AKI in hospitalised patients and its strong association with mortality, observations that are confirmed by our results. However, specific mode of death in this group is rarely reported. The only recent data pertain to a relatively small number of critically ill patients from intensive care settings in whom sepsis is the most commonly reported CoD [9], [10], [17]. A variety of other pathologies were reported to a lesser degree. Studies examining CoD in more generalised AKI populations date back at least two decades and predate current diagnostic criteria. As such, only those with severe renal impairment were studied; in a study by Barretti et al 50% of patients received renal replacement therapy (RRT) whereas Woodrow et al included AKI patients with either a serum creatinine of >600 µmol/l or receiving RRT [8], [11]. Similar to intensive care patients, sepsis was the most common CoD in 38% and 42.5% of patients respectively, with cardiovascular and respiratory disease the next most commonly reported. In the Madrid ARF study, factors contributing to mortality were shock in 46% of cases, infection in 44%, respiratory disease in 22% and cardiovascular disease in 15% [18].

Our study is the first to report CoD in a large number of patients with AKI in a generalised hospital population, with two-thirds of this group having less severe AKI (stage 1). In keeping with other studies, sepsis remained the most common CoD, and this predominantly consisted of pneumonia. Ischaemic heart disease and cardiac failure were also prevalent. These results emphasise the important effect of co-existing conditions on patient outcomes. There is also an increasing evidence base that points towards the crucial effects of organ cross-talk in AKI, in which the development of AKI has a negative impact on distant organ function [12]. It is therefore interesting to note that although only a minority of patients died from uraemia, more than 75% of non-survivors had ongoing AKI at time of death. Work on organ cross talk linking AKI to pathological changes in the lung and myocardium may have particular relevance to the large proportion of patients who died due to pneumonia and cardiac failure [12].

In the absence of specific treatments for AKI, attempts have been made to develop quality improvement strategies to combat the deficiencies that are both common and well documented in the routine clinical care of patients with AKI [13], [19], [20]. To maximise effectiveness, our results suggest that such strategies should focus not just on the management of AKI and its complications but also on the most relevant co-existing conditions. Clearly, any such interventions would need to be tested in appropriate clinical trials, but may include linkage of AKI management into pneumonia or sepsis care bundles, or attempts to prevent the under-treatment of cardiac disease in the setting of renal dysfunction [21], [22]. Equally, our results demonstrate certain high risk groups to which particular attention must be paid; this includes patients with liver disease, heart failure and pneumonia as well as those with hospital-acquired AKI. However, it remains that whatever the associated clinical scenario, the development of AKI is a marker of the unwell patient who is at greater risk of mortality and deserved of increased attention.

In contrast to previous studies in which it was rarely implicated, malignancy was the third most common CoD in our population. There may be several factors contributing to this change over time. It may reflect the increasing age and frailty of patients undergoing chemotherapy who are particularly vulnerable to AKI in the setting of neutropaenic sepsis, fluid depletion or nephrotoxic insults [23]. It is also likely that a proportion of patients developed AKI in the terminal stages of their illness in whom there will not have been a reversible element to their condition. This is consistent with the independent association between mortality and metastatic disease. It is also possible that the inclusion of more patients with less severe AKI had an effect on increasing the proportion of patients with fatal malignancy.

The factors associated with AKI in our population were similar to previously reported, and include age, co-morbid conditions (particularly liver disease, heart failure and malignancy), the severity of AKI and developing AKI during a hospital stay. The severity of AKI retained a strong association with mortality even after the effects of age and co-morbid conditions were considered. However, as we have previously reported, there was less of a difference in mortality rates between AKI stages 2 and 3 in this heterogenous group of AKI patients from a general hospitalised population [15]. This is explained by the inclusion of patients with pre-existing CKD in whom the AKIN diagnostic criteria perform less well; when patients with normal baseline renal function are studied the expected increase in mortality is seen with each increased in AKI stage [15]. This effect of CKD has implications for clinical practice with respect to interpreting the AKIN criteria in this group, and also suggests current diagnostic criteria need further refinement.

The under reporting of AKI on MCCD is a novel observation, but is consistent with the concept that AKI is often under-recognised in routine clinical practice [19]. This may reflect under-recognition of the condition itself or a failure to appreciate the potential impact of the presence of AKI on poor outcomes.

This study does have some potential weaknesses. As an observational study, it is not possible to attribute causality to any of the observed associations. Using hospital coding data to derive co-morbidity does have potential limitations with respect to accuracy, although the use of ICD-10 provides a standardised method to collect relatively detailed clinical information. In addition, we did not have access to details regarding the severity of acute illness or other biochemical variables, which were therefore not included in the analysis of factors associated with mortality. Finally, it must be acknowledged that the CoD derived from MCCD may not always be accurate, as in the absence of a post-mortem examination it is completed according to ‘the best of the doctor’s knowledge and belief’. However, the validity of this approach is supported by a very similar pattern of CoD in the subset of those that underwent post-mortem examination.

In conclusion, we provide a current description of the leading CoD in patients with AKI from a general hospitalised population, as well as important associations with mortality. These data should help inform future strategies to improve outcomes in this high risk group that target not just AKI but the most relevant associated conditions.
